# Absence of Long-Range Magnetic Ordering in a Trirutile High-Entropy Oxide (Mn_0.2_Fe_0.2_Co_0.2_Ni_0.2_Cu_0.2_)Ta_1.92_O_6−δ_

**DOI:** 10.1021/acs.inorgchem.4c04165

**Published:** 2025-02-11

**Authors:** Gina Angelo, Liana Klivansky, Jeremy G. Philbrick, Tai Kong, Jian Zhang, Xin Gui

**Affiliations:** † Department of Chemistry, University of Pittsburgh, Pittsburgh, Pennsylvania 15260, United States; ‡ The Molecular Foundry, Lawrence Berkeley National Laboratory, Berkeley, California 94720, United States; § Department of Physics, University of Arizona, Tucson, Arizona 85721, United States; ∥ Department of Chemistry and Biochemistry, University of Arizona, Tucson, Arizona 85721, United States

## Abstract

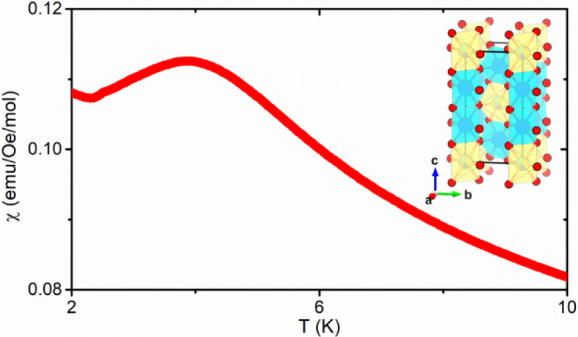

Functionalities of solid-state materials are usually considered to be dependent on their crystal structures. The limited structural types observed in the emerging high-entropy oxides put constraints on the exploration of their physical properties and potential applications. Herein, we synthesized the first high-entropy oxide in a trirutile structure, (Mn_0.2_Fe_0.2_Co_0.2_Ni_0.2_Cu_0.2_)Ta_1.92_O_6−δ_, and investigated its magnetism. The phase purity and high-entropy nature were confirmed by powder X-ray diffraction and energy-dispersive spectroscopy, respectively. X-ray photoelectron spectroscopy indicated divalent Mn, Co, Ni, and Cu along with trivalent Fe. Magnetic property measurements showed antiferromagnetic coupling and potential short-range magnetic ordering below ∼4 K. The temperature-dependent heat capacity data measured under zero and high magnetic fields confirmed the lack of long-range magnetic ordering and a possible low-temperature phonon excitation. The discovery of the first trirutile high-entropy oxide opens a new pathway for studying the relationship between the highly disordered atomic arrangement and magnetic interaction. Furthermore, it provides a new direction for exploring the functionalities of high-entropy oxides.

## Abstract 2

We report the first high-entropy oxide in a trirutile structure. Both powder XRD and EDS measurements confirm the high-entropy nature and the formula, (Mn_0.2_Fe_0.2_Co_0.2_Ni_0.2_Cu_0.2_)Ta_1.92_O_6**−**δ_. Antiferromagnetic spin−spin coupling under high temperatures and short-range antiferromagnetic ordering under low temperatures were observed. The discovery of the first trirutile high-entropy oxide builds a new platform for investigating the interplay between high-entropy nature and its magnetism. Moreover, it allows the manipulation of a wider range of physical properties in high-entropy oxides.

## Introduction

High-entropy oxides (HEOs) are a type of material in which five or more elements are randomly distributed in equiatomic stoichiometry on the same atomic site. Vast applications, such as catalysis^1^ and reversible energy storage,^2^ have been found for HEOs. Better mechanical properties and resistance to oxidation/corrosion have also been observed for HEOs.^3,4^ In addition, magnetic insulating HEOs have been reported due to their potential application in next-generation memory and spintronic devices.^5^ Since Rost et al. discovered the first HEO in a rocksalt structure,^6^ there have been great efforts to expand the structural types of HEOs. To date, ten major crystal structures have been found for HEOs: bixbyite (A_2_O_3_),^7^ delafossite (ABO_2_),^8^ fluorite (AO_2_),^9^ magnetoplumbite (AB_12_O_19_),^10^ mullite-type (A_2_B_4_O_10_),^11^ perovskite (ABO_3_),^12^ pyrochlore (A_2_B_2_O_7_),^13,14^ rocksalt (AO),^6^ Ruddlesden–Popper phase (A_*n*__+1_B_*n*_O_3__*n*__+1_),^15^ and spinel (AB_2_O_4_),^16^ where A and B are cations. However, many structural types remain obscure, which significantly limits the manipulation and exploration of HEOs’ properties. Furthermore, research surrounding their magnetic properties has only recently been studied.^17^ Upon investigation, HEOs can possess complicated magnetic properties due to the disordering of multiple transition metals, e.g., the spin-glass states in perovskite HEOs^18^ and tunable magnetism in spinel HEOs.^16^ Considering the importance of the crystal structure in determining physical properties, expanding the structural families of HEOs is crucial for exploring their functionalities and potential applications.

Herein, we report the first trirutile HEO, (Mn_0.2_Fe_0.2_Co_0.2_Ni_0.2_Cu_0.2_)Ta_1.92_O_6−δ_, belonging to space group *P*4_2_/*mnm* (no. 136), as evidenced by X-ray diffraction (XRD) and energy-dispersive spectroscopy (EDS). While there were reports of rutile HEOs,^19^ the trirutile structure remains unfounded. Trirutile, i.e., AB_2_O_6_, is a derivative of the rutile structure, i.e., AO_2_, wherein an additional element disrupts the repeatability of the rutile structure such that a 1 × 1 × 3 superlattice is formed. NiTa_2_O_6_,^20^ CuTa_2_O_6_^21^, and CoTa_2_O_6_^22^ have all been studied for their antiferromagnetic properties as trirutile oxides, and we employed CoTa_2_O_6_ as the parent compound, which was reported to be antiferromagnetically ordered below T_N_ ∼ 6.6 K.^22^ However, when doped with Mg, i.e., Co_1–*x*_Mg_*x*_Ta_2_O_6_, the antiferromagnetic ground state is quenched at *x* = 10%, while a short-range ferromagnetic correlation can be found in all series of compositions.^23^ Considering that the theoretical calculations suggest a ferromagnetic ground state,^23^ competing ferromagnetic and antiferromagnetic interactions must exist in CoTa_2_O_6_. Our investigation on the physical properties of (Mn_0.2_Fe_0.2_Co_0.2_Ni_0.2_Cu_0.2_)Ta_1.92_O_6−δ_ suggests short-range antiferromagnetic interaction resulting from the highly disordered transition-metal site. Therefore, trirutile CoTa_2_O_6_ provides a good platform for investigating the interplay between magnetism and the crystallographic disorder on the Co site, with further possibilities to extend the scope of physical properties in trirutile HEOs.

## Experimental Details

### Synthesis of (Mn_0.2_Fe_0.2_Co_0.2_Ni_0.2_Cu_0.2_)Ta_1.92_O_6−δ_

(Mn_0.2_Fe_0.2_Co_0.2_Ni_0.2_Cu_0.2_)Ta_1.92_O_6−δ_ was prepared by mixing CoO powder (99.995%, Thermo Scientific), MnO powder (99.99%, Thermo Scientific), CuO powder (99.7%, ∼200 mesh, Alfa Aesar), NiO powder (99.998%, Thermo Scientific), Fe_2_O_3_ powder (99.9%, Thermo Scientific), and Ta_2_O_5_ powder (99.5%, Thermo Scientific), and by placing the 0.2:0.2:0.2:0.2:0.1:0.96 mixture into an alumina crucible. The crucible was covered with a small alumina lid, placed into a larger crucible, and then surrounded with activated charcoal (untreated, ≤5 mm, Sigma-Aldrich). This assembly was then placed into a microwave (1000 W, Model 40GR47) in the air, equipped with firebricks for further insulation, and microwaved for a total of 30 at 5-min intervals with grinding in between. The sample was then pressed into a pellet and placed in a furnace preheated to 1250 °C in air for 1 h, then air quenched. The resulting sample is a homogeneous brown chunk that is stable in air. A high-purity phase could not be realized without a 4% deficiency in Ta_2_O_5_ or microwaving, the reasons for which are detailed later.

### Phase Identification

(Mn_0.2_Fe_0.2_Co_0.2_Ni_0.2_Cu_0.2_)Ta_1.92_O_6−δ_ was crushed into a powder and prepared for powder X-ray diffraction (XRD). A Bruker D2 PHASER instrument was used with Cu Kα radiation (λ = 1.54060 Å, Ge monochromator). The Bragg angle measured was from 5 to 100° at a rate of 1.7°/min with a step of 0.012°. Rietveld fitting in FULLPROF was employed to analyze the crystal structure and test the phase purity of (Mn_0.2_Fe_0.2_Co_0.2_Ni_0.2_Cu_0.2_)Ta_1.92_O_6−δ_.^24^

### Physical Property Measurements

Magnetic properties were measured in a Quantum Design Dynacool Physical Properties Measurement System (PPMS) (1.8–300 K, 0–9 T) equipped with ACMS II, with both DC and AC magnetic fields available, as well as a vibrating-sample magnetometer (VSM). Heat capacity was measured in the PPMS from 1.9 to 37 K. Resistivity measurements were conducted in the PPMS from 310 to 350 K using the four-probe method, with platinum wires attached to the representative sample using silver epoxy.

### Scanning Electron Microscopy (SEM) with Energy-Dispersive Spectroscopy (EDS)

Compositional analysis was performed via scanning electron microscopy (SEM) with energy-dispersive spectroscopy (EDS). A Zeiss Sigma 500 VP SEM instrument with Oxford Aztec X-EDS was used with an electron beam energy of 20 kV.

## Results and Discussion

### Crystal Structure and Compositional Analysis

**Figure 1:**
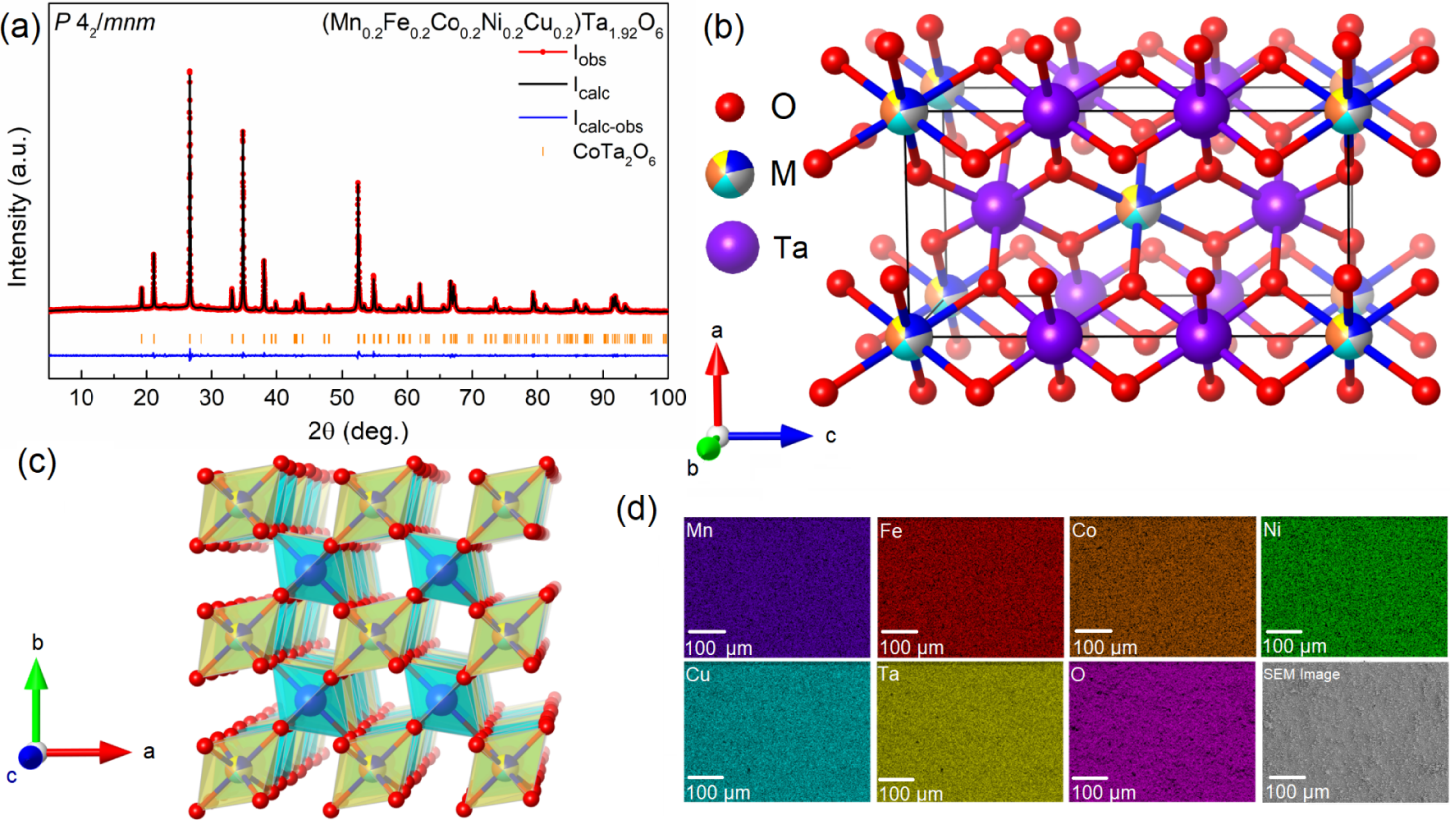
(a) Powder XRD pattern of (Mn_0.2_Fe_0.2_Co_0.2_Ni_0.2_Cu_0.2_)Ta_1.92_O_6−δ._ The observed pattern is shown by the red line with dots, while the black line represents the calculated pattern. The difference between the observed and calculated pattern is shown in blue. Bragg peak positions of CoTa_2_O_6_ are visualized with orange vertical bars. (b) (Mn_0.2_Fe_0.2_Co_0.2_Ni_0.2_Cu_0.2_)Ta_1.92_O_6−δ_ trirutile structure is viewed from the *b* axis. Here, “M” stands for the 3*d* transition metal in (Mn_0.2_Fe_0.2_Co_0.2_Ni_0.2_Cu_0.2_)Ta_1.92_O_6−δ_. (c) (Mn_0.2_Fe_0.2_Co_0.2_Ni_0.2_Cu_0.2_)Ta_1.92_O_6−δ_ viewing from the *c* axis with Ta@O_6_ (cyan) and M@O_6_ (yellow) octahedra. (d) SEM-EDS elemental mapping results of Co, Cu, Fe, Mn, Ni, Ta, and O on a ∼0.11 mm^2^ area of the pellet sample.

Powder XRD pattern of the air-quenched sample from 5° to 100° is shown in Figure 1a. The pattern was fitted via the Rietveld method by using the crystal structure of reported CoTa_2_O_6_.^25^ The fitting parameters, i.e., *R*_p_= 3.62%, *R*_wp_ = 4.63%, *R*_exp_ = 4.02%, and χ^2^ = 1.32, showed a good match between the trirutile CoTa_2_O_6_ and the proposed HEO (Mn_0.2_Fe_0.2_Co_0.2_Ni_0.2_Cu_0.2_)Ta_1.92_O_6**–**δ_. No prominent secondary phase was found, indicating that (Mn_0.2_Fe_0.2_Co_0.2_Ni_0.2_Cu_0.2_)Ta_1.92_O_6−δ_ crystallizes in the same space group as CoTa_2_O_6_, i.e., *P*4_2_/*mnm* with high purity. The refined lattice parameters of (Mn_0.2_Fe_0.2_Co_0.2_Ni_0.2_Cu_0.2_)Ta_1.92_O_6−δ_ are *a* = 4.73179(1) Å and *c* = 9.20395(3) Å, which creates a slightly smaller unit cell than CoTa_2_O_6_ (*a* = 4.73580 Å, *c* = 9.17080 Å) with smaller tetragonality, i.e., a smaller c/a ratio. The fitted atomic coordinates are shown in Table S1 which further confirms the trirutile structure for (Mn_0.2_Fe_0.2_Co_0.2_Ni_0.2_Cu_0.2_)Ta_1.92_O_6−δ._ The obtained crystal structure is shown in Figure 1b,c. Edge-shared M@O_6_ and Ta@O_6_ octahedra stack along the *c* axis and are connected to each other in a corner-sharing manner. The M@O_6_ layers with a high-entropy site consisting of 3*d* transition metals within the *ab* plane are separated by Ta@O_6_ layers.

In order to confirm the high-entropy nature of (Mn_0.2_Fe_0.2_Co_0.2_Ni_0.2_Cu_0.2_)Ta_1.92_O_6−δ_, an SEM-EDS analysis was performed. In two distinct regions of (Mn_0.2_Fe_0.2_Co_0.2_Ni_0.2_Cu_0.2_)Ta_1.92_O_6−δ_, elemental maps were collected and can be seen in Figure 1d, for which the results are shown in Table S2. The average chemical formula was found to be Mn_0.19(1)_Fe_0.20(1)_Co_0.18(1)_Ni_0.20(1)_Cu_0.20(2)_Ta_1.92(1)_O_6−δ_, displaying near equiatomic ratios of each 3*d* transition metal. Given that each 3*d* transition metal M can form a stable phase in MTa_2_O_6_,^21,22,26,27^ our material is not an entropy-stabilized material.^6^ Meanwhile, we note that there is a deficiency in oxygen based on EDS and further examined this in the magnetic superexchange pathway section. Since (Mn_0.2_Fe_0.2_Co_0.2_Ni_0.2_Cu_0.2_)Ta_1.92_O_6−δ_ is an insulator (as discussed later), charging effects may affect the EDS results.^28^ When considering this, our results are within the error of the loading composition. To study the surface composition of our sample, XPS was performed. Three points of interest were analyzed to determine the surface concentration of the 3*d* transition metals to be 14(5)% Mn, 35(4)% Fe, 27(8)% Co, 10(2)% Ni, and 14(10)% Cu. As exemplified by the high levels of deviation as well as the nonequiatomic ratios, there are surface inhomogeneities in the sample. More analysis of XPS is provided in the SI. XPS has a penetration depth of ∼10 nm,^29^ while EDS has a penetration depth of around 2 μm at 20 keV.^30^ Therefore, the surface inhomogeneities will less likely affect the bulk properties. Since our studies focus on the bulk properties of (Mn_0.2_Fe_0.2_Co_0.2_Ni_0.2_Cu_0.2_)Ta_1.92_O_6–δ_, the EDS-determined chemical composition will reflect more precisely the bulk composition. Therefore, all subsequent calculations were performed based on the loading composition, i.e., (Mn_0.2_Fe_0.2_Co_0.2_Ni_0.2_Cu_0.2_)Ta_1.92_O_6–δ_. Further information about XPS results can be found in the Supporting Information.

### Short-Range Antiferromagnetic Ordering in (Mn_0.2_Fe_0.2_Co_0.2_Ni_0.2_Cu_0.2_)Ta_1.92_O_6−δ_

**Figure 2:**
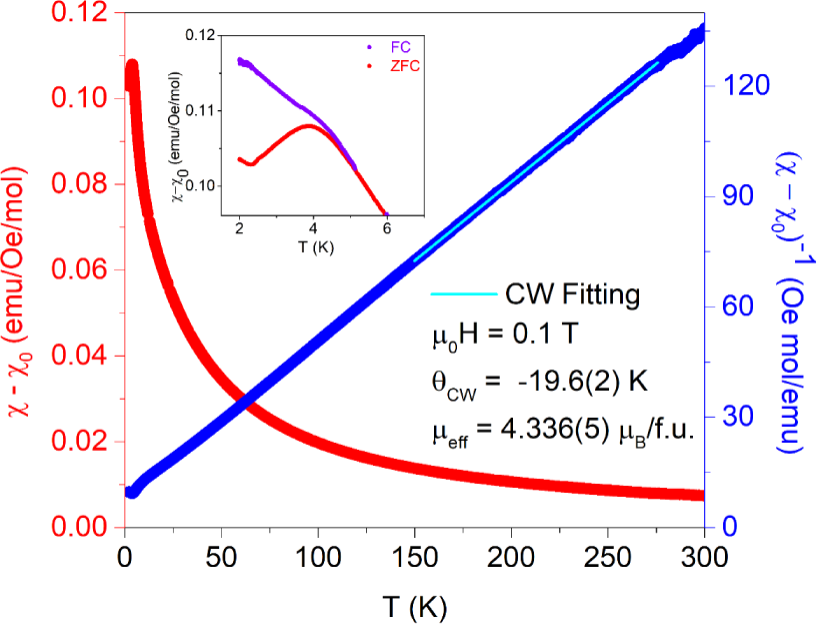
Magnetic susceptibility minus χ_0_ (left red axis) and inverse of magnetic susceptibility minus χ_0_ (right blue axis) of (Mn_0.2_Fe_0.2_Co_0.2_Ni_0.2_Cu_0.2_)Ta_1.92_O_6−δ_ measured under an external magnetic field of 0.1 T with respect to temperature. The cyan line represents the Curie–Weiss fitting. The inset zooms in on the difference between the inverse of magnetic susceptibility minus χ_0_ with respect to temperature under zero-field cooling (red line) and field cooling (purple line) at an external magnetic field of 0.1 T.

(Mn_0.2_Fe_0.2_Co_0.2_Ni_0.2_Cu_0.2_)Ta_1.92_O_6−δ_ was measured for the temperature dependence of the magnetic susceptibility from 1.8 to 300 K under an external magnetic field of 0.1 T in a zero-field cooling mode, as shown in Figure 2. Paramagnetic behavior was seen under high temperature, while a broad peak can be observed at ∼4 K, as discussed later. Curie–Weiss (CW) fitting was performed from 150 to 275 K according to the modified CW law:
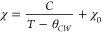
where χ is the magnetic susceptibility, *C* is the Curie constant independent of temperature and related to the effective moment (*μ_eff_*), *θ_CW_* is the CW temperature, and χ_0_ is a constant independent of temperature and related to the core diamagnetism and temperature-independent paramagnetic contributions such as Pauli paramagnetism. The χ_0_ parameter was added to CW fitting since a slightly positive curvature was noted at ∼250 K and was fitted to be 0.00109(1) emu/Oe/mol. The resulting *θ*_*CW*_ is −19.6 (2) K, indicating antiferromagnetic (AFM) spin–spin coupling within the fitted temperature region. Therefore, the slight deviation from CW behavior at ∼58 K might originate from the onset of AFM coupling, as seen in Figure S4. The resulting *θ_CW_* is enhanced in comparison to the parent Mn (−4.2 K),^26^ Fe (−8.11(5) K),^27^ Co (−6.63(5) K),^22^ Ni (−10.3(2) K),^22^ and Cu (−1.5(2) K)^21^ tantalate compounds.

Based on *μ_eff_* (spin-only) =  where *n* is the number of unpaired electrons, the spin-only moment for all 3*d* transition metal ions except for Co^2+^ in a high-spin octahedral crystal field in our material is as follows: Mn^2+^ (∼5.92 μ_B_), Fe^3+^ (∼5.92 μ_B_), Ni^2+^ (∼2.83 μ_B_), and Cu^2+^ (∼1.73 μ_B_). Since Co^2+^ ions exhibit large single-ion anisotropy, we chose to use the effective moment reported for CoTa_2_O_6_, 4.9(2) μ_B_ for Co^2+^ instead.^23^ The *μ_eff_* when considering equal contributions from each 3d transition metal, averages to ∼4.26 μ_B_, which is consistent with the *μ_eff_* from CW fitting determined via  to be 4.336 (5) μ_B_/f.u. The larger fitted *μ_eff_* might be due to the unaccounted contributions from the orbital angular momentum.

As shown in Figure 2 inset, the χ vs T curve features a broad downward parabola shape at ∼4 K when under zero-field cooling (ZFC), unlike the more linear shape that is realized under field-cooling (FC). Therefore, we performed the temperature-dependent AC magnetic susceptibility measurements with a DC field of 100 Oe and an AC field of 10 Oe under different frequencies to confirm the existence of a spin-glass state. As shown in Figure S5, no frequency-dependent behavior can be seen for the AC susceptibility under 936 Hz, 1635 Hz, and 1858 Hz from 2 to 10 K, which excludes a conventional spin-glass state in (Mn_0.2_Fe_0.2_Co_0.2_Ni_0.2_Cu_0.2_)Ta_1.92_O_6−δ_. Meanwhile, the DC magnetic susceptibility under the FC protocol does not reach a plateau in our material, which further confirms the lack of a conventional spin-glass state. The divergence between the ZFC and FC curves below ∼4 K normally originates from spin freezing.^31,32^ Therefore, the broad peak observed in Figure 2 might originate from short-range antiferromagnetic ordering, which will be further discussed later along with the heat capacity results. Moreover, similar behavior can be seen in Mg-doped CoTa_2_O_6_ where short-range magnetic ordering was observed when the Mg concentration is above ∼10%.^23^

**Figure 3:**
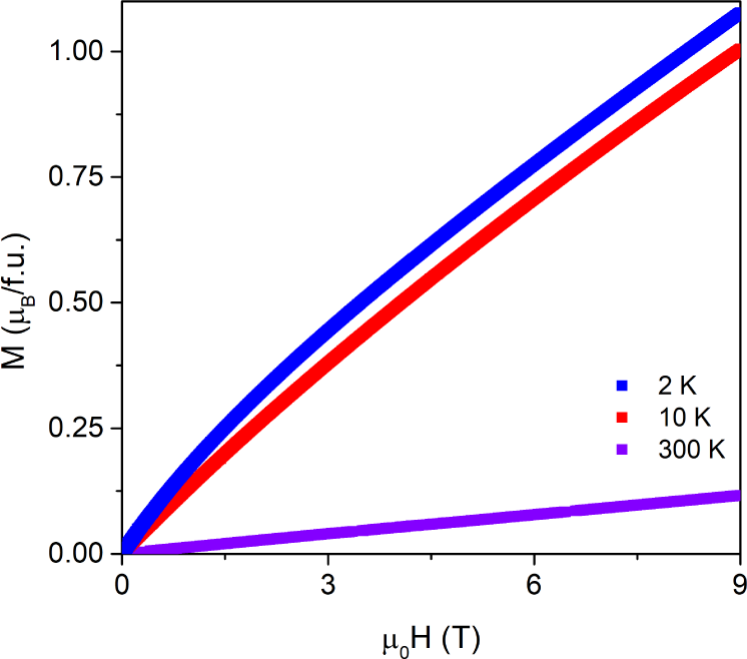
Field-dependent magnetization from 0 to 9 T at 2, 10, and 300 K, in blue, red, and violet, respectively.

The field dependence of magnetization, as shown in Figure 3, illustrates that at 2, 10, and 300 K, (Mn_0.2_Fe_0.2_Co_0.2_Ni_0.2_Cu_0.2_)Ta_1.92_O_6−δ_ does not become saturated up to 9 T. A slightly bent feature can be seen at 2 K, which is typical for the paramagnetic state. In addition, no coercive field is observed, as can be seen in Figure S6, which can be indicative of the absence of a long-range ordered ferromagnetic component.

### Heat Capacity

**Figure 4:**
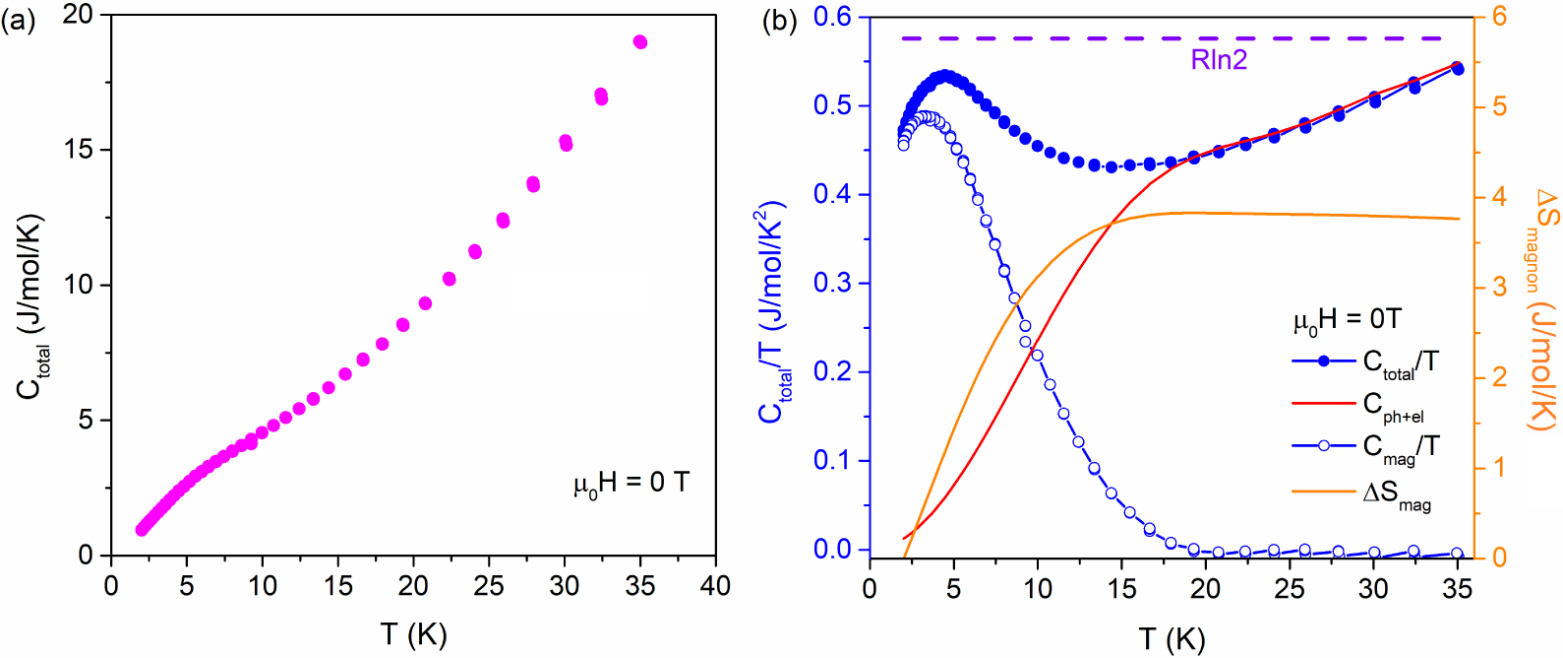
(a). *C*_total_ is shown in magenta as a function of the temperature. (b) *C*_total_/*T* is shown in blue filled circles while *C*_mag_/*T* is shown in blue open circles. The fitted *C*_ph_ is graphed in red. These three curves correspond to the left blue axis. Rln2 is shown by the orange dashed line, and Δ*S*_mag_ is the solid orange line; both lines correspond to the right orange axis.

The temperature-dependent heat capacity of (Mn_0.2_Fe_0.2_Co_0.2_Ni_0.2_Cu_0.2_)Ta_1.92_O_6−δ_ was measured from 2 to 35 K. No indication of long-range magnetic ordering, i.e., a λ-anomaly in the heat capacity curve, can be seen, as shown in Figure 4a. While a broad feature appears at ∼4 K in Figure 4a, Figure 4b plots the *C*_total_/*T* vs *T* curve, and the broad peak is shown at ∼4 K. The total heat capacity of a magnetic material can be treated as the sum of electronic, phononic, and magnonic contributions where *C*_total_ = *C*_el_ + *C*_ph_ + *C*_mag_. As shown by resistivity in Figure S7 (Mn_0.2_Fe_0.2_Co_0.2_Ni_0.2_Cu_0.2_)Ta_1.92_O_6**–**δ_ exhibits insulating behavior; thus, there are no electronic contributions to heat capacity, i.e., *C*_total_ = *C*_ph_ + *C*_mag_. As an estimate, *C*_ph_ = *βT*^*3*^ according to the Debye formula, where β is the vibrational contribution coefficient. However, just a *T*^3^ term could not lead to a good fitting. This could be due to unaccounted high-frequency phononic contributions to heat capacity, as the Debye formula is only an estimate. Herein, *T*^5^, *T*^7^, *T*^9^, and *T*^11^ terms were added to obtain a reasonable fitting so that a polynomial fit was employed: *C*_ph_/*T* = β_1_*T*^2^ + β_2_*T*^4^ + β_3_*T*^6^ + β_4_*T*^8^ + β_5_*T*^10^ where β_1_, β_2_, β_3_, β_4_, and β_5_ are constants and were fitted to 0.00311(9) J/mol/K^4^, −8.6(5) × 10^–6^ J/mol/K^6^, 1.2(1) × 10^–8^ J/mol/K^8^, −7.7(8) × 10^–12^ J/mol/K^10^, and 2.0(2) × 10^–15^ J/mol/K^12^. Therefore, by subtracting the phononic contribution from *C*_total_/*T*, pure C_mag_/*T* is plotted in Figure 4b as blue open circles. By integrating the *C*_mag_/*T* vs *T* curve, the change in magnetic entropy, Δ*S*_mag_, is obtained. Δ*S*_mag_ is usually determined by the spin multiplicity in a magnetic system where contributions from orbital angular momentum can be ignored by Δ*S*_mag_ = Rln(2*S*+1), where R is the gas constant. Here, Δ*S*_mag_ does not exceed Rln2, which is the magnetic entropy change for an *S* = 1/2 system. Because the average spin state of the 3*d* transition metals appearing in (Mn_0.2_Fe_0.2_Co_0.2_Ni_0.2_Cu_0.2_)Ta_1.92_O_6−δ_, i.e., Mn^2+^ (*S* = 5/2), Fe^3+^ (*S* = 5/2), Co^2+^ (*S* = 3/2), Ni^2+^ (*S* = 1), and Cu^2+^ (*S* = 1/2), must be larger than *S* = 3/2. Therefore, the observed Δ*S*_mag_ is obviously smaller than that of Rln4, indicating the absence of long-range magnetic ordering within the measured temperature range in (Mn_0.2_Fe_0.2_Co_0.2_Ni_0.2_Cu_0.2_)Ta_1.92_O_6−δ_. The heat capacity of (Mn_0.2_Fe_0.2_Co_0.2_Ni_0.2_Cu_0.2_)Ta_1.92_O_6−δ_ was also measured under an external magnetic field, as shown in Figure S8; no shift is observed for peak position. The field-independent behavior suggests that such an anomaly below ∼5 K is not spin-related; instead, it can be due to phononic excitation.

### Magnetic Superexchange Interaction and Possible Origin of Short-Range Magnetic Ordering

**Figure 5:**
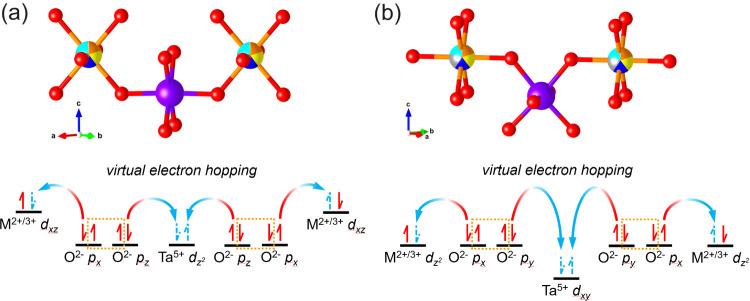
Two different magnetic superexchange pathways in (Mn_0.2_Fe_0.2_Co_0.2_Ni_0.2_Cu_0.2_)Ta_1.92_O_6−δ_ between transition metal ions. M^2+/3+^ stands for transition metal ions. The relative vertical distance between atomic orbitals stands for the relative geometry of ions. The dashed orange lines involve the pair of electrons on difference p orbitals of oxygen ions that stabilize the superexchange pathways by inclusion of exchange energy.

Based on the crystal structure of (Mn_0.2_Fe_0.2_Co_0.2_Ni_0.2_Cu_0.2_)Ta_1.92_O_6**–**δ_, two possible magnetic superexchange pathways can be proposed, as illustrated in Figure 5, while the other pathways are related to them by symmetries. These superexchange pathways are based on the Goodenough-Kanamori rule^33,34^ and the atomic orbitals of M^2+/3+^ near the Fermi energy are determined by the semiempirical extended-Hückel-tight-binding (EHTB) methods and CAESAR packages, as described in the Supporting Information. The calculation was only conducted on the parent compound, CoTa_2_O_6_, due to the complexities of the high-entropy (Mn_0.2_Fe_0.2_Co_0.2_Ni_0.2_Cu_0.2_)Ta_1.92_O_6−δ_. However, the symmetries of molecular orbitals in CoTa_2_O_6_ can be well extended to (Mn_0.2_Fe_0.2_Co_0.2_Ni_0.2_Cu_0.2_)Ta_1.92_O_6−δ_ regardless of the addition or removal of valence electrons of 3*d* transition metal ions. Therefore, the analysis of molecular orbitals in the high-entropy compound can be conducted based on the highest-occupied molecular orbital in CoTa_2_O_6_ by varying the number of electrons.

The first superexchange pathway is shown in Figure 5a where two M@O_6_ octahedra are connected by a Ta@O_6_ octahedron in an axial manner. In this case, a half-filled d_*xz*_ orbital of M^2+/3+^, fully filled p_*x*_ and p_*z*_ orbitals of O^2–^ and an empty d_*z*_^2^ orbital of Ta^5+^ are present. The two M^2+/3+^ ions on the opposite sides show antiferromagnetic coupling for the following reasons: 1. Due to the virtual electron hopping represented by the arrows, a pair of spins with opposite spin directions from oxygen p_*z*_ orbitals can populate the empty d_*z*_^2^ orbital of Ta^5+^, which provides a more stable ground state. 2. The exchange energies between the spins with the same direction on p_*x*_ and p_*z*_ orbitals of O^2–^ stabilize the system as well, as indicated by the orange dashed lines in Figure 5 3. Virtual electron hopping can also occur between the p_*x*_ orbitals of O^2–^ and the half-filled d_*xz*_ orbital of M^2+/3+^, which leads to a stable singlet state. The superexchange interaction depicted results in antiferromagnetic coupling between the M^2+/3+^ ions. Similar reasons and superexchange patterns can be found in the second pathway, shown in Figure 5b, while the involved atomic orbitals are now the half-filled d_*z*_^2^ orbital of M^2+/3+^, fully filled p_*x*_ and p_*y*_ orbitals of O^2–^ and an empty d_*xy*_ orbital of Ta^5+^. In both cases, antiferromagnetic coupling between M ions is dominant.

With the analysis above, we can speculate on three possible reasons for the short-range magnetic ordering observed in magnetic properties measurements:

- The existence of Ni^2+^ and Cu^2+^ leads to the lack of half-filled d_*xz*_ orbitals, which disables the scenario in Figure 5a where a half-filled d_*xz*_ orbital of M^2+/3+^ is necessary. Subsequently, the corresponding superexchange pathway is not functional due to the lack of electron hopping and, thus, magnetic coupling is absent. Meanwhile, all the M^2+/3+^ ions possess a half-filled d_*z*_^2^ orbital, which allows the superexchange interaction in Figure 5b. Therefore, with part of the superexchange pathways disabled, long-range magnetic ordering does not exist in (Mn_0.2_Fe_0.2_Co_0.2_Ni_0.2_Cu_0.2_)Ta_1.92_O_6−δ_.
- Due to the existence of Fe^3+^, in order to maintain charge neutrality, possible removal of cations can take place as a result of ionic charge compensation mechanism. Therefore, part of the M^2+^ or Ta^5+^ sites may be vacant, leading to the interruption of the superexchange pathways and the absence of long-range magnetic ordering. The Ta^5+^ vacancy is seen in the inability to synthesis a high-purity sample with nondeficient Ta_2_O_5_ amounts. In addition, to maintain charge neutrality, there must be oxygen deficiencies that similarly disrupt the superexchange pathway.
- The high-entropy nature of the M^2+/3+^ site can result in the local distortion of the M@O_6_ octahedra, which reduces the orbital overlap between M^2+/3+^ and the intermediate O^2–^. Consequently, electron hopping may not occur due to the altered symmetries of orbitals.

## Conclusion

In this article, we report the first high-entropy oxide in a trirutile structure. A high-purity phase with high configurational disorder in a uniform structure has been determined via powder XRD and EDS measurements. The new material, (Mn_0.2_Fe_0.2_Co_0.2_Ni_0.2_Cu_0.2_)Ta_1.92_O_6−δ_, shows antiferromagnetic spin–spin coupling at high temperatures while exhibiting short-range antiferromagnetic ordering at low temperatures. Heat capacity further shows evidence of the absence of long-range magnetic ordering in this compound. XPS shows the presence of divalent Mn, Co, Ni, and Cu, along with trivalent Fe, which are consistent with the observations in the magnetic results. The discovery of the first trirutile high-entropy oxide builds a new platform for investigating the interplay between the high-entropy nature and its magnetism. Moreover, it allows for the manipulation of a wider range of physical properties in high-entropy oxides.

## Supporting Information Available

Deviation from Curie–Weiss behavior below ∼50 K. AC magnetic susceptibility data. Magnetic hysteresis loop. Temperature-dependent resistivity data. Heat capacity data under zero and applied magnetic field. Powder XRD fitted atomic coordinates. EDS results (PDF)
