# Supplementary material for: Absence of Long-Range Magnetic Ordering in a Trirutile High-Entropy Oxide (Mn0.2Fe0.2Co0.2Ni0.2Cu0.2)Ta1.92O6−δ
Source: Inorg Chem. 2025 Feb 11;64(7):3196–202. doi: 10.1021/acs.inorgchem.4c04165 (PMC11863364; doi:10.1021/acs.inorgchem.4c04165)
Supplement: Supplementary file 1 — ic4c04165_si_001.pdf [file ic4c04165_si_001.pdf]

Supporting Information

Absence of Long-Range Magnetic Ordering in a Trirutile High-Entropy Oxide $(Mn_{0.2}Fe_{0.2}Co_{0.2}Ni_{0.2}Cu_{0.2})Ta_{1.92}O_{6-\delta}$

Gina Angelo,[a] Liana Klivansky,[b] Jeremy G. Philbrick,[c] Tai Kong,[c,d] Jian Zhang,[b] Xin Gui [a]*

[a] Department of Chemistry, University of Pittsburgh, Pittsburgh, PA, 15260, USA
[b] The Molecular Foundry, Lawrence Berkeley National Laboratory, Berkeley, CA, 94720, USA
[c] Department of Physics, University of Arizona, Tucson, AZ, 85721, USA
[d] Department of Chemistry and Biochemistry, University of Arizona, Tucson, AZ, 85721, USA

Table of Contents

Table S1 2

Table S2 2

Figure S1 3

Table S3 4

Figure S2 5

Table S4 6

Figure S3 7

Table S5 7

Figure S4 10

Figure S5 11

Figure S6 12

Figure S7 13

Figure S8 14

Table S1. The atomic sites of $(Mn_{0.2}Fe_{0.2}Co_{0.2}Ni_{0.2}Cu_{0.2})Ta_{1.92}O_{6-\delta}$ were determined via Rietveld refinement. $(Mn_{0.2}Fe_{0.2}Co_{0.2}Ni_{0.2}Cu_{0.2})Ta_{1.92}O_{6-\delta}$ is in space group $P4_2/mnm$ (no. 136) with parameters a = 4.73179 (1) Å and c = 9.20395 (3) Å. M stands for 3*d* transition metals.

	x	***y***	***z***
**M1**	0	0	0
**Ta1**	0	0	0.3317 (1)
**O1**	0.2896 (9)	0.2896 (9)	0
**O2**	0.3007 (6)	0.3007 (6)	0.3337 (6)

Table S2 EDS results of $MTa_{1.92}O_6$ for two different areas of the sample. Data was normalized to Ta and compared to the loading composition.

Spectra	Mn	Fe	Co	Ni	Cu	Ta
Map 1	2.18	2.33	2.13	2.27	2.46	22.07
Map 2	2.30	2.31	2.09	2.33	2.16	22.18
Average	2.24 (8)	2.32 (2)	2.11 (2)	2.30 (4)	2.31 (21)	22.13 (7)
Normalized to Ta as 1.92	0.19 (1)	0.20 (1)	0.18 (1)	0.20 (1)	0.20 (2)	1.92 (1)
Loading Composition	0.2	0.2	0.2	0.2	0.2	1.92

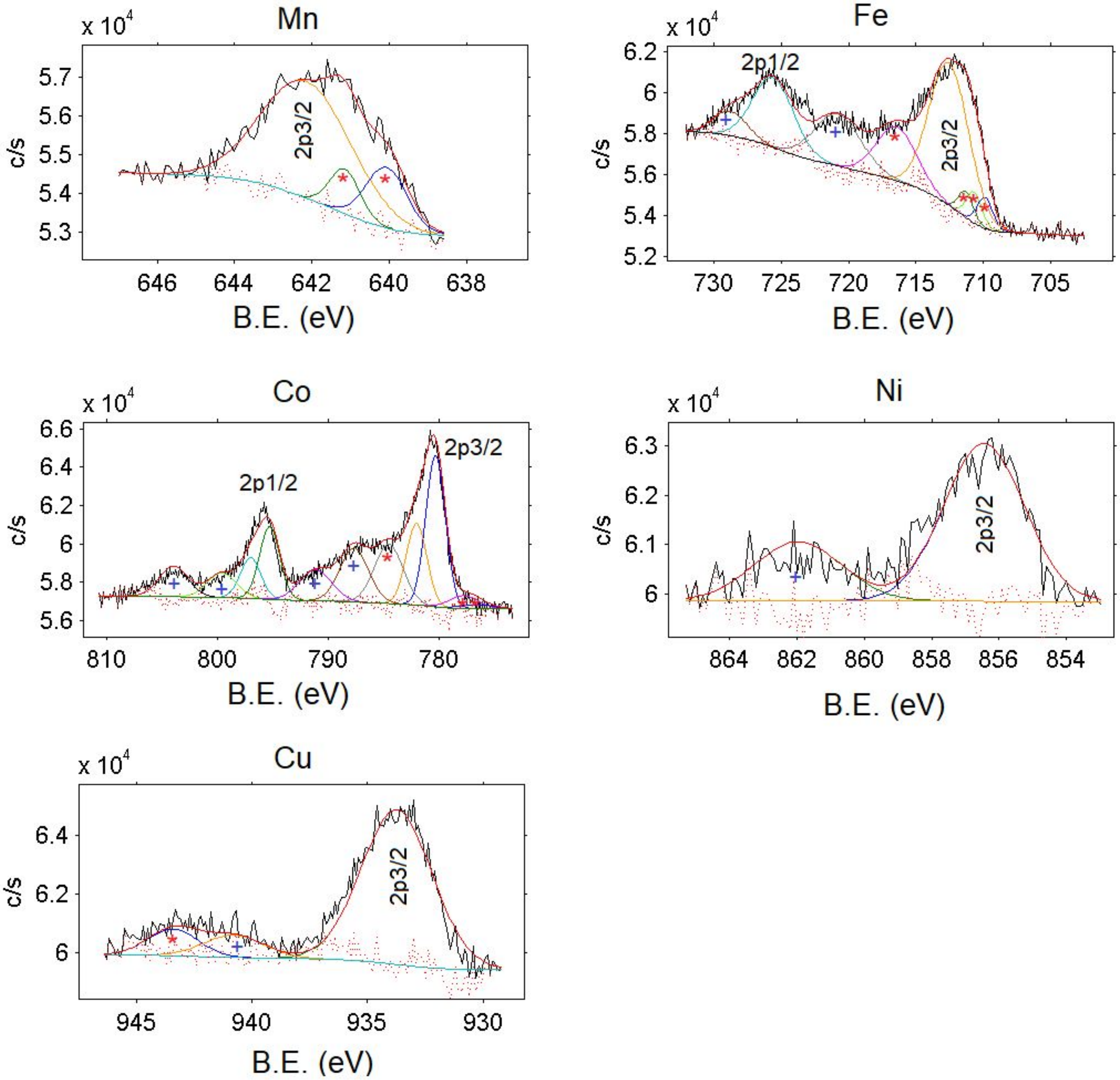

Figure S1. Peak fitting of Mn, Fe, Co, Ni, and Cu in $(Mn_{0.2}Fe_{0.2}Co_{0.2}Ni_{0.2}Cu_{0.2})Ta_{1.92}O_{6-\delta}$ in area 1. B.E. stands for binding energy. Blue plus signs mark satellite peaks wile red asterisks mark Auger peaks.

Table S3. Peak fitting of Mn, Fe, Co, Ni, and Cu regions in $(Mn_{0.2}Fe_{0.2}Co_{0.2}Ni_{0.2}Cu_{0.2})Ta_{1.92}O_{6-\delta}$ in area 1.

Element/ Transition	Peak Energy (eV)	Peak FWHM (eV)	Peak Area (eV*cts/s)	Peak Assignment	RSF	Atomic %
Cu LMM	640.07	1.27	2124	Cu Auger		
Ni LMM	641.16	0.92	1145	Ni Auger		
Mn 2p3/2	642.14	2.74	8835	Mn^{2+}	1.757	14.38
Ni LMM	709.85	1.40	2254	Ni Auger		
Cu LMM	710.68	1.40	2254	Cu Auger		
Co LMM	711.28	1.31	1793	Co Auger		
Fe 2p3/2	712.53	3.49	25885	Fe^{3+}	2.946	37.58
Cu LMM	716.45	3.52	9841	Cu Auger		
Fe 2p Sat	720.82	4.05	10933	Satellite		
Fe 2p1/2	725.63	3.57	12943	Fe^{3+}		
Fe2p sat	728.82	2.88	3684	Satellite		
Co LMM	776.58	3.58	758	Co Auger		
Ni LMM	777.79	3.08	2177	Ni Auger		
Co 2p3/2	780.32	2.08	17520	Co^{2+}	3.529	12.04
Co 2p3/2	782.04	2.20	9975	Co^{3+}	3.529	20.41
Fe LMM	784.55	3.08	10108	Fe Auger		
Co 2p sat	787.67	3.08	9230	Satellite		
Co 2p sat	791.16	3.46	5907	Satellite		
Co 2p1/2	795.29	2.16	8760	Co^{2+}		
Co2p1/2	797.01	2.20	4988	Co^{3+}		
Co 2p sat	799.60	3.08	4216	Satellite		
Co 2p sat	803.96	3.08	5244	Satellite		
Ni 2p3/2	856.45	2.82	9621	Ni^{2+}	2.309	12.30
Ni 2p3 sat	861.97	3.04	3861	Satellite		
Cu 2p3/2	933.73	3.26	20323	Cu^{2+}	2.626	3.29
Cu 2p sat	940.79	3.06	2462	Satellite		
Mn LMM	943.43	2.56	2474	Mn Auger		

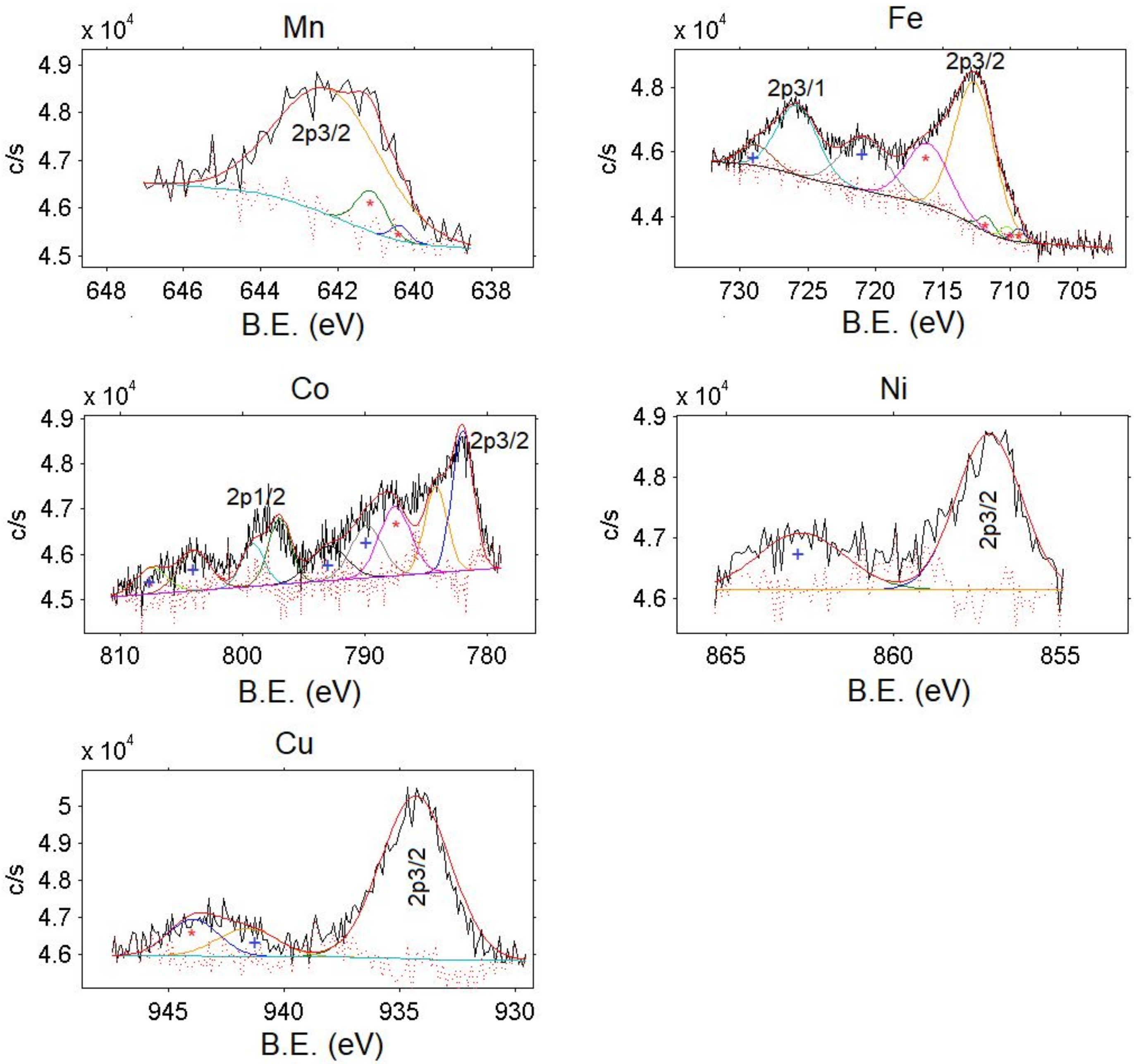

Figure S2. Peak fitting of Mn, Fe, Co, Ni, and Cu in $(Mn_{0.2}Fe_{0.2}Co_{0.2}Ni_{0.2}Cu_{0.2})Ta_{1.92}O_{6-\delta}$ in area 2. B.E. stands for binding energy. Blue plus signs mark satellite peaks wile red asterisks mark Auger peaks.

Table S4. Peak fitting of Mn, Fe, Co, Ni, and Cu regions in $(Mn_{0.2}Fe_{0.2}Co_{0.2}Ni_{0.2}Cu_{0.2})Ta_{1.92}O_{6-\delta}$ in area 2.

Element/ Transition	Peak Energy (eV)	Peak FWHM (eV)	Peak Area (eV*cts/s)	Peak Assignment	RSF	Atomic %
Cu LMM	640.38	0.56	193	Cu Auger		
Ni LMM	641.13	0.91	828	Ni Auger		
Mn 2p3/2	642.23	3.20	8980	Mn^{2+}	1.757	18.89
Ni LMM	709.37	1.15	403	Ni Auger		
Cu LMM	710.2	1.15	492	Cu Auger		
Co LMM	711.75	1.31	677	Co Auger		
Fe 2p3/2	712.66	3.49	16372	Fe^{3+}	2.946	30.71
Cu LMM	716.10	3.78	7633	Cu Auger		
Fe 2p3Sat	720.82	4.05	7005	Satellite		
Fe 2p1/2	725.76	3.57	8186	Fe^{3+}		
Fe2p sat	728.82	2.78	1859	Satellite		
Co 2p3/2	781.98	2.07	6827	Co^{2+}	3.529	10.65
Co 2p3/2	784.18	2.20	4455	Co^{3+}	3.529	6.95
Fe LMM	787.53	3.08	4993	Fe Auger		
Co 2p sat	789.83	3.08	3788	Satellite		
Co 2p sat	792.98	3.58	2909	Satellite		
Co 2p1/2	796.95	2.20	3413	Co3O4		
Co2p1/2	799.15	2.20	2228	Co3O4		
Co 2p sat	803.94	3.08	2904	Satellite		
Co 2p sat	807.43	3.08	1807	Satellite		
Ni 2p3	857.15	2.27	6231	Ni^{2+}	2.309	9.85
Ni 2p3 sat	862.76	3.04	3030	Satellite		
Cu 2p3/2	934.30	3.56	16632	Cu^{2+}	2.626	22.96
Cu 2p sat	941.59	3.06	2453	Satellite		
Mn LMM	943.94	2.56	2712	Mn Auger		

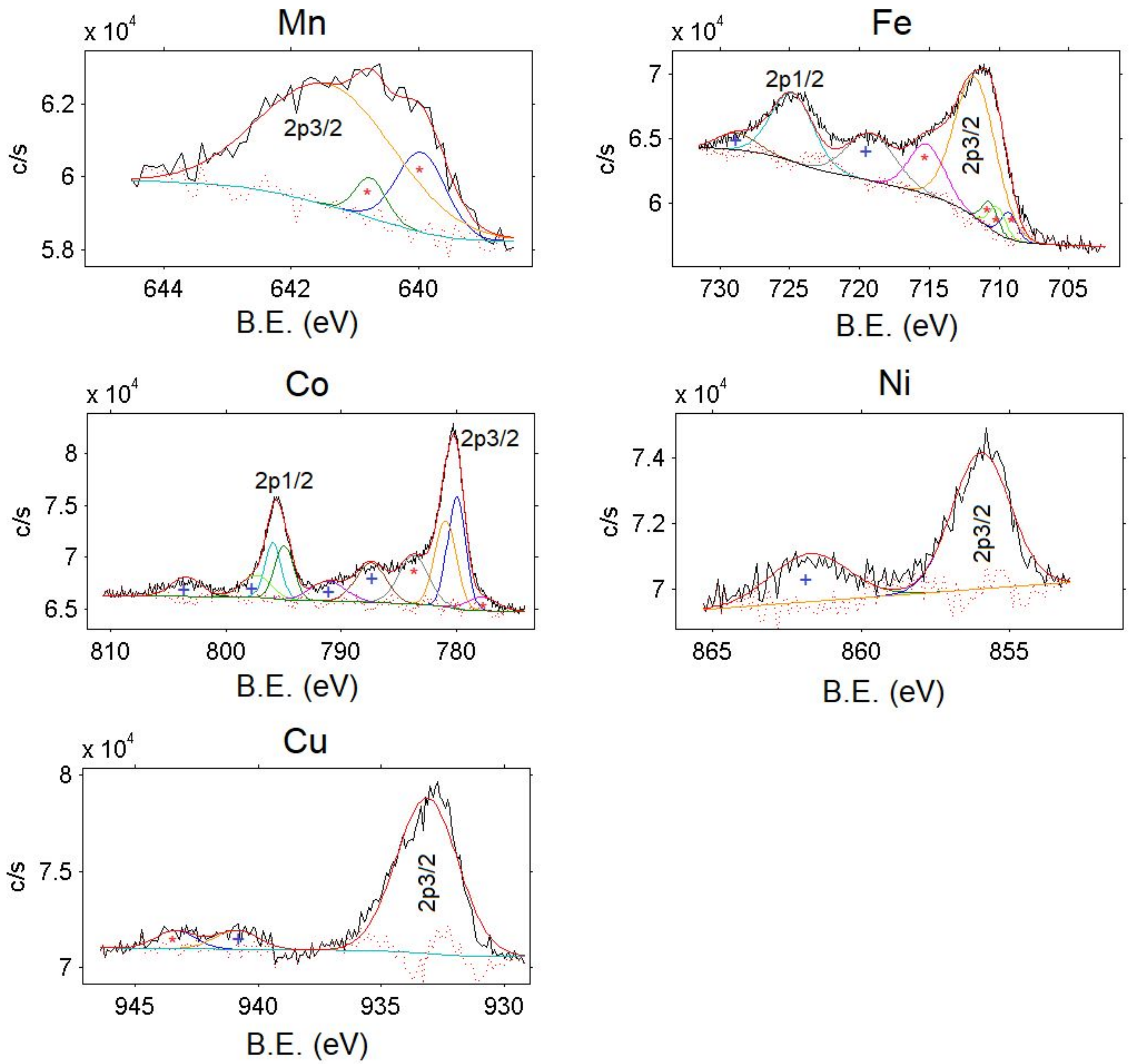

Figure S3. Peak fitting of Mn, Fe, Co, Ni, and Cu in $(Mn_{0.2}Fe_{0.2}Co_{0.2}Ni_{0.2}Cu_{0.2})Ta_{1.92}O_{6-\delta}$ in area 3. B.E. stands for binding energy. Blue plus signs mark satellite peaks wile red asterisks mark Auger peaks.

Table S5. Peak fitting of Mn, Fe, Co, Ni, and Cu regions in $(Mn_{0.2}Fe_{0.2}Co_{0.2}Ni_{0.2}Cu_{0.2})Ta_{1.92}O_{6-\delta}$ in area 3.

Element/Transition	Peak Energy (eV)	Peak FWHM (eV)	Peak Area (eV*cts/s)	Peak Assignment	RSF	Atomic (%)
Cu LMM	639.95	0.91	2134	Cu Auger		
Ni LMM	640.75	0.56	659	Satellite		
Mn 2p3/2	641.42	2.49	8822	Mn Auger		
Cu LMM	639.95	0.91	2134	Cu Auger		
Ni LMM	640.75	0.56	659	Ni Auger		
Mn 2p3/2	641.42	2.49	8822	Mn^{2+}	1.757	8.90
Ni LMM	709.29	1.4	3024	Ni Auger		
Cu LMM	710.12	1.4	3024	Cu Auger		
Co LMM	710.67	1.31	2832	Co Auger		
Fe 2p3/2	711.72	3.49	40272	Fe^{3+}	2.946	36.23
Cu LMM	714.97	3.02	11908	Cu Auger		
Fe 2p Sat	719.18	4.05	15814	Satellite		
Fe 2p1/2	724.82	3.57	20136	Fe^{3+}		
Fe2p sat	728.82	2.78	1859	Satellite		
Ni LMM	777.79	3.08	4287	Ni Auger		
Co 2p3/2	779.98	1.85	21280	Co^{2+}	3.529	15.92
Co 2p3/2	780.95	2.20	19342	Co^{3+}	3.529	14.47
Fe LMM	783.72	3.08	15241	Fe Auger		
Co 2p sat	787.42	3.08	12465	Satellite		
Co 2p sat	791.16	3.58	7264	Satellite		
Co 2p1/2	794.95	1.93	10640	Co^{2+}		
Co2p1/2	795.92	1.68	9671	Co^{3+}		
Co 2p sat	797.38	3.08	6975	Satellite		
Co 2p sat	803.47	3.08	6114	Satellite		
Ni 2p3	855.97	2.31	10212	Ni^{2+}	2.309	7.74
Ni 2p sat	861.73	3.04	4714	Satellite		
Cu 2p3/2	933.14	2.94	25269	Cu^{2+}	2.626	16.74
Cu 2p sat	940.87	2.06	2164	Satellite		
Mn LMM	943.35	1.86	1836	Mn Auger		

Oxidation States Analysis:

X-ray photoelectron spectroscopy (XPS) was performed in a ULVAC-PHI Inc. PHI GENESIS instrument equipped with monochromatic Al K_α radiation (1486.6 eV) as the excitation source. The X-ray analysis area for measurement was set at 100 μm diameter and a flood gun (1.6 V, 20 μA) was used for charge compensation. The pass energy was 112 eV for the high-resolution regions. The analysis chamber pressure was less than 1×10^{-7} mbar during data acquisition. Fittings were performed with the MultiPak software. C1s C-C was used as a reference at 284.8eV for all spectra. Smart backgrounds were used to fit all elements. Constraints such as peak separation and area ratio confined to 2:1 for doublet peaks were used. Due to the high degree of transition metals with overlapping auger peaks, the peak fitting was nontrivial. For Mn, Ni and Cu, only the 2p3/2 peak area was fitted since they had no overlap with peaks outside the fitted region.

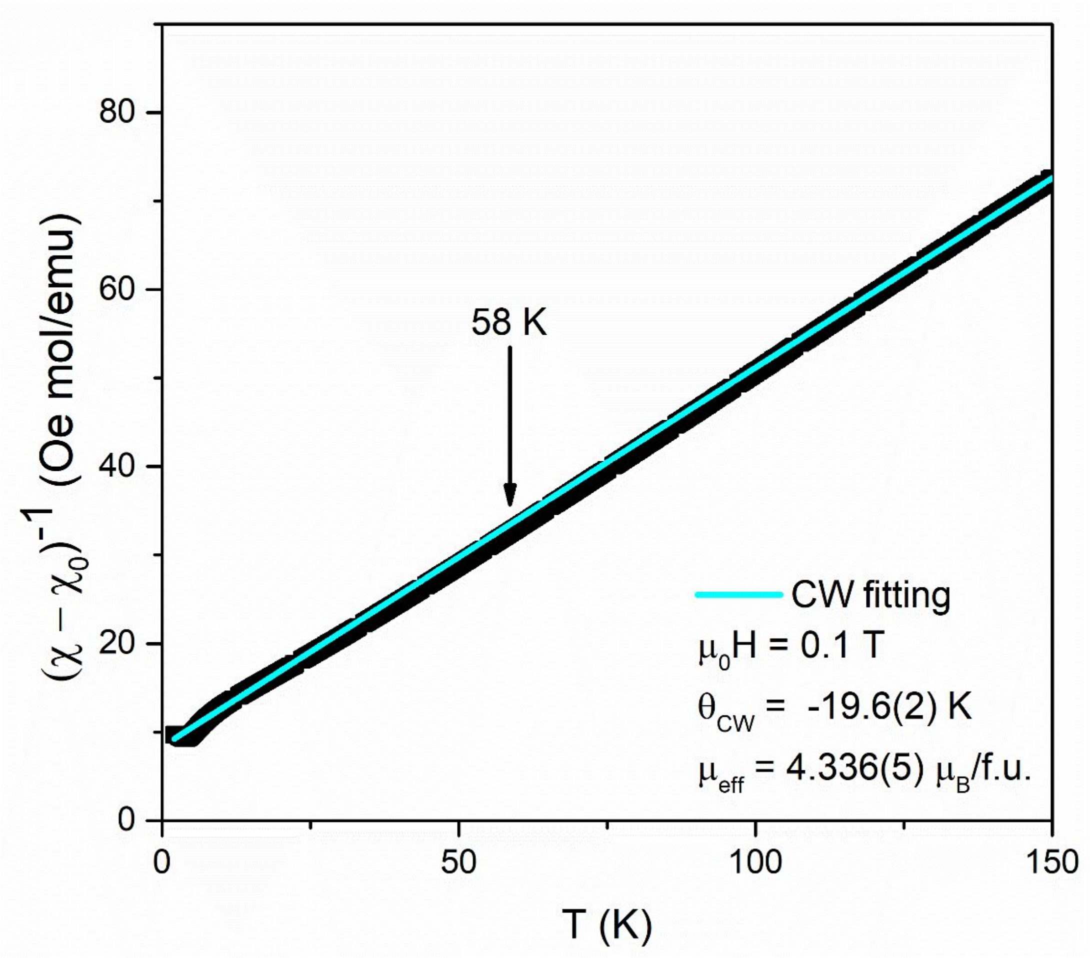

Figure S4. Deviation from the Curie-Weiss (CW) behavior at low temperatures.

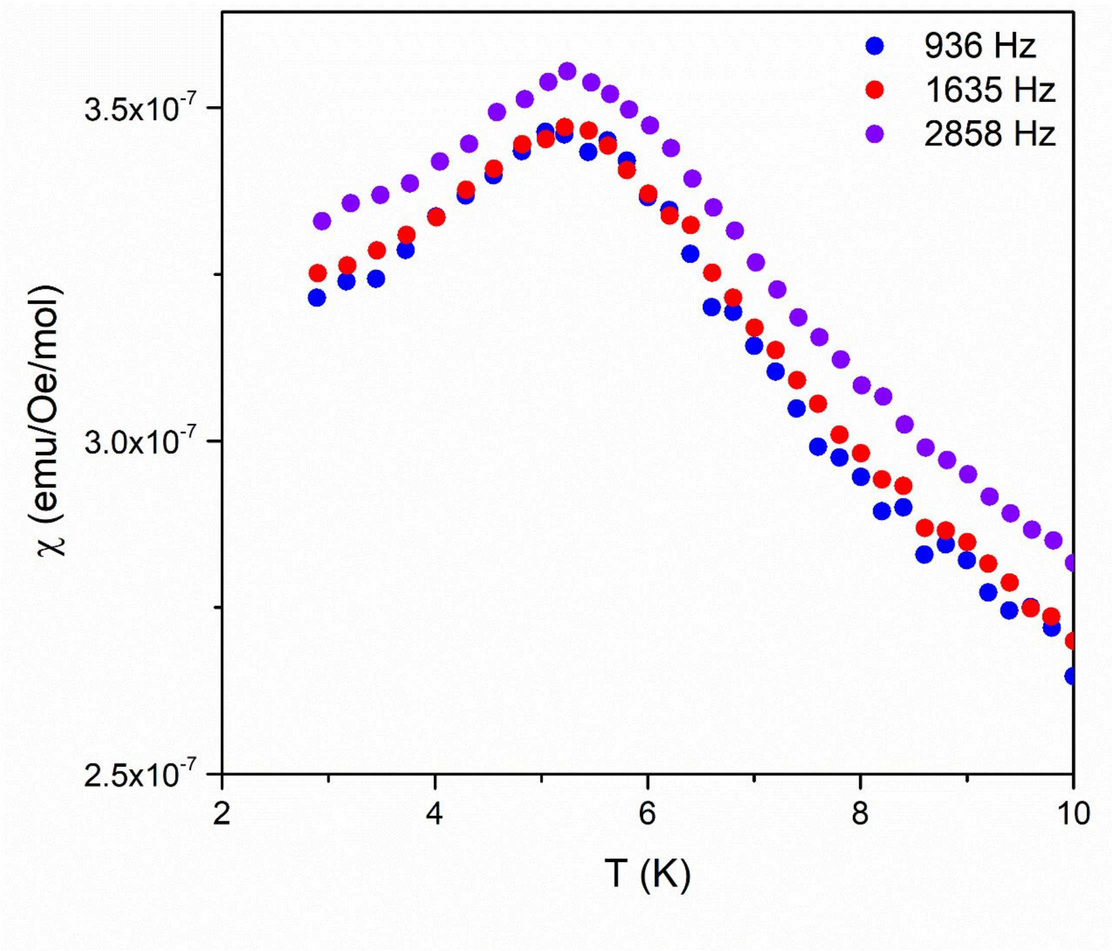

Figure S5. AC magnetic susceptibility of $(Mn_{0.2}Fe_{0.2}Co_{0.2}Ni_{0.2}Cu_{0.2})Ta_{1.92}O_{6-\delta}$ under various frequencies. The applied DC magnetic field is 100 Oe and the AC field is 10 Oe.

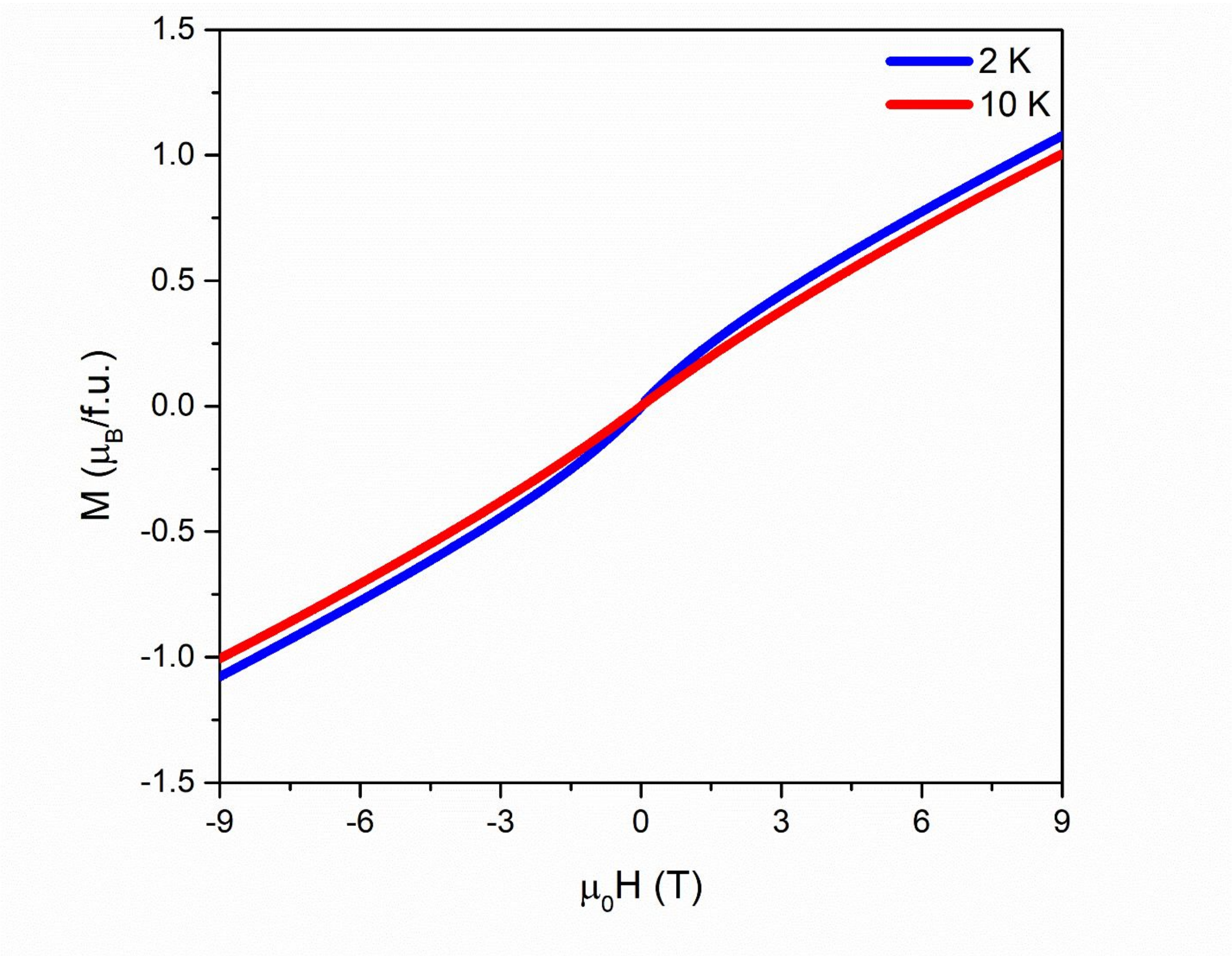

Figure S6. Hysteresis loops of $(Mn_{0.2}Fe_{0.2}Co_{0.2}Ni_{0.2}Cu_{0.2})Ta_{1.92}O_{6-\delta}$ at 2 K (blue) and 10 K (red).

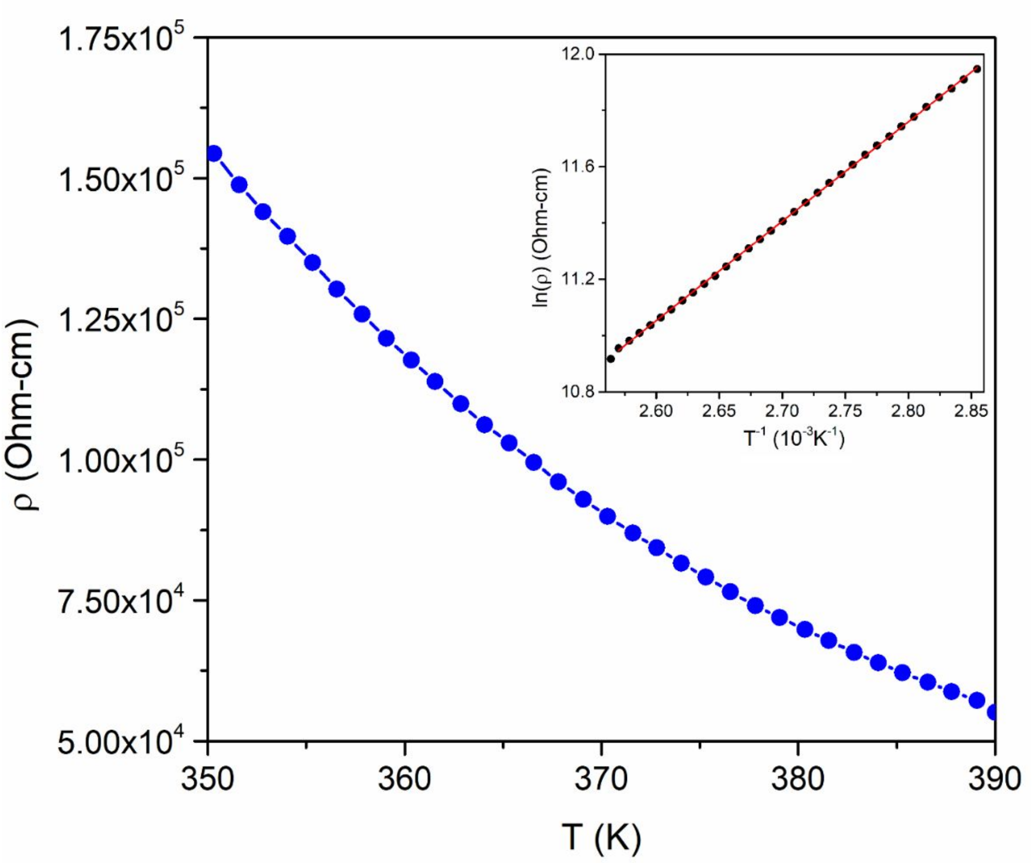

Figure S7. Resistivity of $(Mn_{0.2}Fe_{0.2}Co_{0.2}Ni_{0.2}Cu_{0.2})Ta_{1.92}O_{6-\delta}$ from 350 K to 390K displaying insulating behavior. The inset graph is of the linear relationship between ln(ρ) and T^{-1}. To calculate the electronic bandgap (E_g), the Arrhenius equation $\rho = \rho_0 e^{E g / 2k_B T}$, where ρ_0 is the pre-exponential term constant and $k_B$is Boltzmann's constant, was used. E_g was determined to be 608(1) meV.

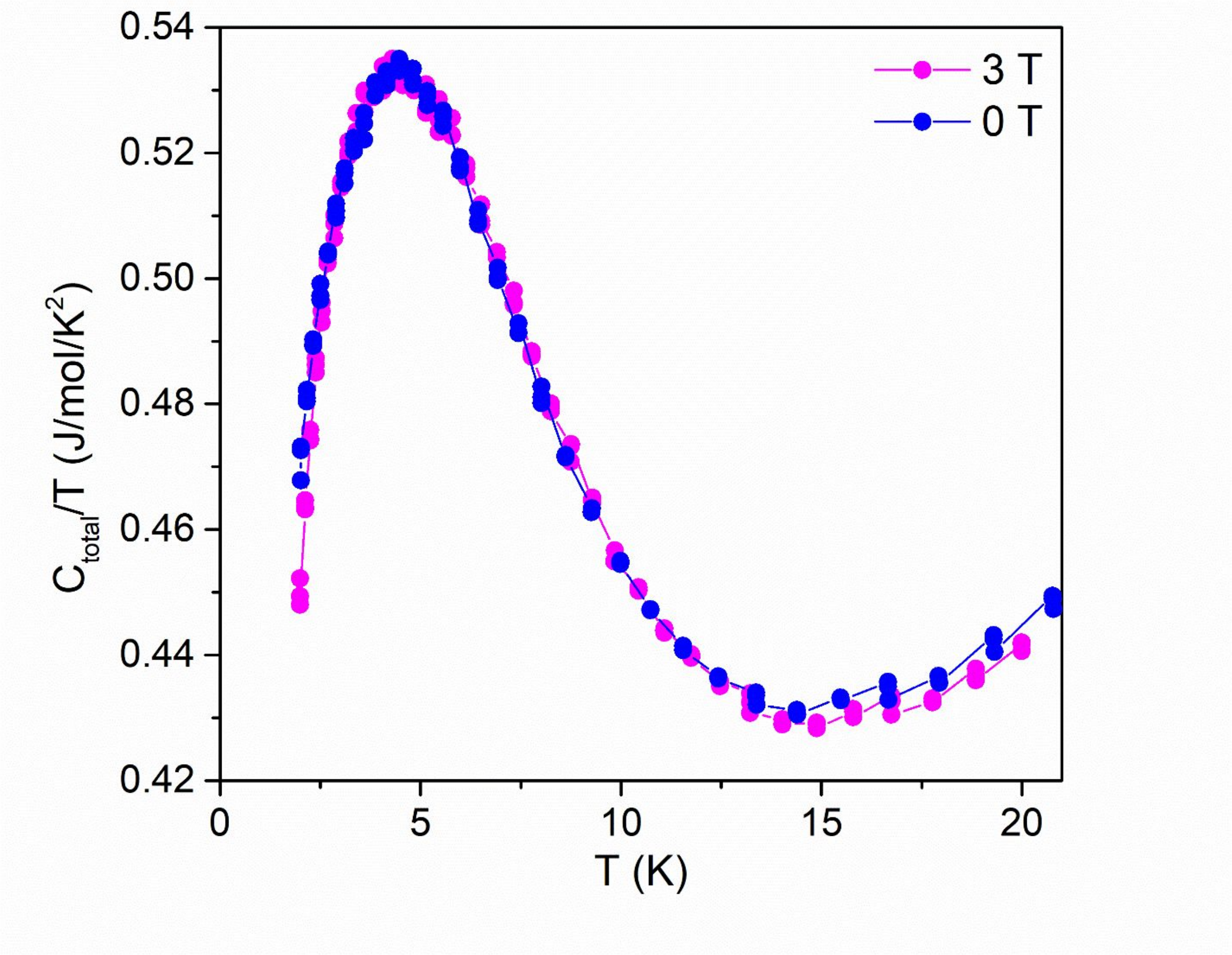

Figure S8. Temperature-dependent heat capacity of $(Mn_{0.2}Fe_{0.2}Co_{0.2}Ni_{0.2}Cu_{0.2})Ta_{1.92}O_{6-\delta}$ measured under magnetic field $\mu_0H = 0$ T and 3 T.

Molecular Orbital (MO) Calculation: Semi-empirical extended-Hückel-tight-binding (EHTB) methods and CAESAR packages are used in calculating molecular orbitals of the parent compound, $CoTa_2O_6$.[1] The basis sets for Co are: 4*s*: Hii = -9.21 eV, ζ1 = 2, coefficient1 = 1.0000; 4*p*: Hii = -5.29 eV, ζ1 = 2, coefficient1 = 1.000; 3*d*: Hii = -13.18 eV, ζ1 = 5.55, coefficient1 = 0.568, ζ2 = 2.1, coefficient2 = 0.606. For Ta: 6*s*: Hii = -10.1 eV, ζ1 = 2.28, coefficient1 = 1.000; 6*p*: Hii = -6.86 eV, ζ1 = 2.241, coefficient1 = 1.000; 5*d*: Hii = -12.1 eV, ζ1 = 4.762, coefficient1 = 0.6815, ζ2 = 1.938, coefficient2 = 0.5589. For O: 2*s*: Hii = -32.29999 eV, ζ1 = 2.275, coefficient1 = 1.000; 2*p*: Hii = -14.8 eV, ζ1 = 2.275, coefficient1 = 1.000.

Reference

1. Hoffmann, R. An extended Hückel theory. I. hydrocarbons. *J. Chem. Phys.* **1963**, *39*, 1397-1412.
